# A Conversation with Alexandra Navrotsky

**DOI:** 10.1021/acscentsci.4c02126

**Published:** 2024-12-30

**Authors:** Rachel Brazil

Alexandra Navrotsky knows a lot about the little things. She is a leader in the field of nanogeoscience, which centers around how nanoparticles lead to mineral formation in geological systems. In over half a century of research, she has made major contributions in mantle mineralogy, deep earth geophysics, and the thermodynamics behind mineral formation. She even lent her name to a recently discovered mineral—navrotskyite.

**Figure:**
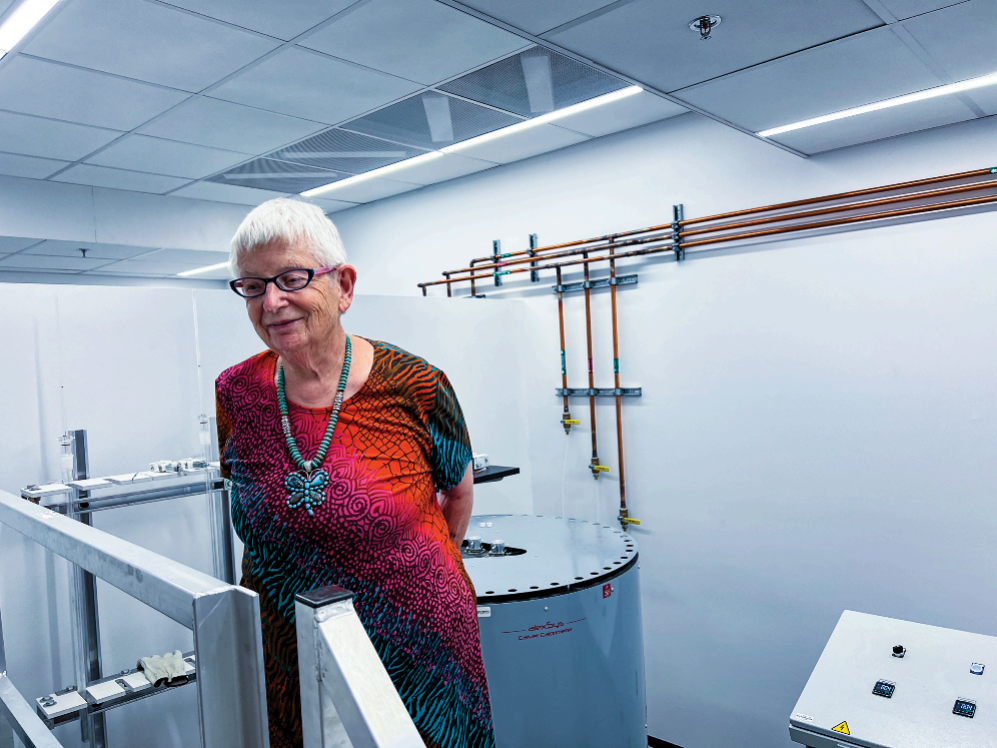
Credit: Alexandra Navrotsky

Now Navrotsky heads up the Navrotsky Eyring Center for Materials of the Universe at Arizona State University. The center represents a unique multidisciplinary initiative aiming to better understand what materials make up other planets, how the planets have formed, and how they evolve over time. Navrotsky is also exploring how materials form in the kinds of extreme conditions found in the universe, using ASU’s new facilities for high-pressure and high-temperature materials research.

Rachel Brazil talked with Navrotsky about the importance of looking at planets on the nanoscale, what we already know about the universe’s many materials, and what she is still hoping to discover. This interview was edited for length and clarity.

## What is nanogeoscience, and why is it important for understanding the chemistry of other planets?

If you think of how many atoms make up a nanometer, it’s about four or five atoms in a row. So nanogeoscience focuses on the behavior of atoms at that scale.

From the geologic and essentially planetary point of view, much, if not almost all, of the reactivity that occurs in the universe occurs with the involvement of small particles. Big particles, big single crystals are really chemically pretty unreactive, and once they are reactive, they will corrode, form small particles, etc. So the majority of reactions—whether we're talking about our solar system, whether we’re talking exoplanets—most of those reactions involve small particles.

Now what is interesting about it is that the small particles have very different properties from large particles. They will be different in their chemical reactivity; they will be different in their thermodynamic properties; they will be different in how they absorb impurities and how they catalyze chemical reactions.

## What do we know about materials on other planets?

One has a general picture of the likely compositions [of elements on other planets], but if one looks at the variety of exoplanets, or one even looks at Mars or Jupiter, then one knows much less about the fundamental chemistry. We know the range of pressures because those are constrained by the size of the planets, and we make educated guesses about the temperatures.

[We have] quite a bit of information now from the [Mars] rovers and of course the various planetary probes. But that information has to be brought together with what the likely chemistry is and what the reactions are or could be. Right now we have more ideas than facts.

## You’re the director of the Materials of the Universe Center at Arizona State University. What is the objective of that center, and what do you hope to achieve?

[We] are addressing the fundamental question of, how do the intrinsic properties of materials determine the evolution of planets, the properties of planets, the properties of resources?

Venus, Earth, and Mars: They’re similar in size; they’re similar in containing a lot of silicate minerals. But they’re very different in how those minerals react; they’re very different in what their surfaces are; they’re very different in how much water and oxygen they contain; therefore, they’re very different in how their properties are influenced by nanoparticles.

The fascinating thing is to try to bring together what we’ve observed in terms of planetary atmospheres and a lot of things that we think we understand about how planets formed, and then look at this at a nanoscopic scale to understand the actual chemistry of what has gone on and what will go on. And that gets us into evolution because if you think of the way planets have changed since their formation, since the formation of the Earth, and since the eventual evolution that led to life, this is all chemistry.

We’re [also] interested in extracting resources from the Earth and eventually from other places in space, perhaps even bringing an asteroid to Earth for its metal content. Maybe there are planets out there that are made entirely of metals; maybe there are planets out there that are made entirely of sulfides.

## ASU recently received a $14.7 million US National Science Foundation grant to set up a facility for high-pressure and high-temperature materials research. What can studying materials in these conditions tell us?

The biggest unanswered question is why materials compress and change their structures the way they do. What is that telling you about how atoms interact? That’s the fundamental physics and chemistry question.

The fundamental materials and geological question is, how do these reactions determine what materials form? And finally, can you harness this richness of pressure and temperature to make new materials? For example, there have been some indications that high pressures can make some interesting new superconducting materials.

High pressure got started, of course, because one wanted it to make diamonds. Well, now one makes diamonds by all sorts of other methods, not just by high pressure.

So similarly, if we find some materials at high pressure that really have interesting properties, then the next step always is going to be to find easier, more economical, more scalable ways of making them. But first you have to discover new materials at high pressure and temperature.

## The mineral navrotskyite was recently discovered in Utah. How did it get named after you?

A mineral is typically named after somebody who has done something significant, perhaps related to that mineral. The second part is you need to have somebody that has identified and characterized a mineral and wants to name it after you. It’s a long proposition through the International Mineralogical Association.

[It] came as an absolute surprise to me. You don’t go through life thinking you will get a mineral named after you. You go through life doing the science that you find fascinating, that you can get funding for. I’m as surprised as anybody that my career has taken me as far as it has.

## Rachel Brazil is a freelance contributor to

Chemical & Engineering News, *the independent news outlet of the American Chemical Society.*

